# Intrinsic Molecular Proton Sensitivity Underlies GPR4 Effects on Retrotrapezoid Nucleus Neuronal Activation and CO_2_-Stimulated Breathing

**DOI:** 10.1523/JNEUROSCI.0799-24.2024

**Published:** 2024-08-06

**Authors:** Elizabeth C. Gonye, Yingtang Shi, Keyong Li, Rachel T. Clements, Wenhao Xu, Douglas A. Bayliss

**Affiliations:** ^1^Department of Pharmacology, University of Virginia, Charlottesville, Virginia 22903; ^2^Genetically Engineered Mouse Model Core, University of Virginia, Charlottesville, Virginia 22903

**Keywords:** brainstem, chemosensation, GPR4, homeostasis, interoception, respiration

## Abstract

An interoceptive homeostatic reflex monitors levels of CO_2_/H^+^ to maintain blood gas homeostasis and rapidly regulate tissue acid–base balance by driving lung ventilation and CO_2_ excretion—this CO_2_-evoked increase in respiration is the hypercapnic ventilatory reflex (HCVR). Retrotrapezoid nucleus (RTN) neurons provide crucial excitatory drive to downstream respiratory rhythm/pattern-generating circuits, and their activity is directly modulated by changes in CO_2_/H^+^. RTN neurons express GPR4 and TASK-2, global deletion of which abrogates CO_2_/H^+^ activation of RTN neurons and the HCVR. It has not been determined if the intrinsic pH sensitivity of these proton detectors is required for these effects. We used CRISPR/Cas9 genome editing to generate mice with mutations in either of two pH-sensing histidine residues in GPR4 to determine effects on RTN neuronal CO_2_/H^+^ sensitivity and the HCVR. In global GPR4(H81F) and GPR4(H167F) mice, CO_2_-stimulated breathing and CO_2_-induced RTN neuronal activation were strongly blunted, with no effect on hypoxia-stimulated breathing. In brainstem slices from GPR4(H81F) mice, peak firing of RTN neurons during bath acidification was significantly reduced compared with GPR4 wild-type mice, and a subpopulation of RTN neurons was rendered pH-insensitive, phenocopying previous results from GPR4-deleted mice. These effects were independent of changes in RTN number/distribution, neuronal excitability or transcript levels for GPR4 and TASK-2. CO_2_-stimulated breathing was reduced to a similar extent in GPR4(H81F) and TASK-2-deleted mice, with combined mutation yielding no additional deficit in the HCVR. Together, these data demonstrate that the intrinsic pH sensitivity of GPR4 is necessary for full elaboration of the HCVR.

## Significance Statement

Among the critical mechanisms for whole-body homeostasis, the hypercapnic ventilatory reflex (HCVR) regulates lung ventilation in order to maintain physiological levels of arterial PCO_2_ and acid–base balance. GPR4 is a proton-activated receptor and putative molecular proton sensor in retrotrapezoid nucleus (RTN) neurons, which are a crucial neural component of this respiratory reflex. In this work, we developed multiple lines of mice in which the intrinsic pH sensitivity of GPR4 was globally disabled by mutations in key histidine residues; in those mice, CO_2_/H^+^ sensitivity of RTN neurons and the HCVR were strongly blunted. These data support the role of GPR4 as a direct molecular sensor for CO_2_/H^+^ in support of this important homeostatic reflex.

## Introduction

Central control of breathing in mammals requires integration of various state-dependent inputs, feedback from central and peripheral chemosensors that monitor blood gases and acid–base status, and activity of brainstem rhythm- and pattern-generating circuits to drive ventilatory motor output (Del Negro et al., 2018). Dysregulation of breathing can be a cause or a symptom of several pathologies including sleep apnea, sudden infant death syndrome, chronic obstructive pulmonary disorder, and congenital central hypoventilation syndrome; these disorders are often accompanied by blunted respiratory responses to changes in blood gases, including CO_2_ (Kepron and Cherniack, 1973; Eckert et al., 2007; Weese-Mayer et al., 2010; Garcia et al., 2013). The physiological mechanisms that contribute to CO_2_-stimulated breathing (hypercapnic ventilatory reflex, HCVR) have been sought for many decades, with varying degrees of success in satisfying key criteria required to implicate specific cells as central respiratory chemoreceptors, including identification of their relevant molecular CO_2_/H^+^ detectors (Gonye and Bayliss, 2023).

Among current chemoreceptor candidates, retrotrapezoid nucleus (RTN) neurons are activated by elevated CO_2_ and provide direct excitatory input to respiratory circuits that increases the frequency and depth of breathing (Mulkey et al., 2004; reviewed in Guyenet and Bayliss, 2022; Gonye and Bayliss, 2023). In mice, the RTN comprises ∼700 neurons located on the ventral medullary surface in close proximity to the facial motor nucleus that are defined by a common developmental lineage (i.e., Egr2-, Phox2b-, Lbx1-, and Atoh1-expressing neurons from the dB2 domain of rhombomere 5; Isik and Hernandez-Miranda, 2022) and neurochemical phenotype (i.e., Phox2b-expressing, glutamatergic neurons that express neuromedin B, NMB; Stornetta et al., 2006; Shi et al., 2017). They express two proton-sensitive molecules that are necessary for CO_2_ modulation of RTN neuronal activity and the HCVR—the proton-activated G-protein–coupled receptor GPR4 and the proton-inactivated potassium channel TASK-2 (Gestreau et al., 2010; Wang et al., 2013; Kumar et al., 2015). Global genetic deletion of either TASK-2 or GPR4 in mice reduces CO_2_/H^+^-stimulated RTN neuronal activation and blunts CO_2_-stimulated breathing, while elimination of both TASK-2 and GPR4 nearly abolishes the HCVR (Gestreau et al., 2010; Wang et al., 2013; Kumar et al., 2015); reexpression of GPR4 in the RTN in the context of a global GPR4 knock-out is sufficient to completely rescue the deficit in CO_2_-stimulated RTN activation and the HCVR (Kumar et al., 2015). These observations suggest that GPR4 and TASK-2 may be the molecular proton detectors that confer intrinsic CO_2_/H^+^ sensitivity on RTN neurons that is relevant for full expression of the HCVR.

In addition to this evidence of their direct CO_2_/H^+^ sensitivity, however, RTN neurons also receive modulatory inputs from various other putative respiratory chemosensory cells, including purinergic activation from local astrocytes and serotonergic facilitation from the caudal raphe (Mulkey et al., 2006, 2007; Ptak et al., 2009; Gourine et al., 2010; Hodges and Richerson, 2010; Wenker et al., 2010, 2012; Sobrinho et al., 2014; Wu et al., 2019; Gonye and Bayliss, 2023). It has been proposed that a majority of the CO_2_/H^+^ sensitivity of RTN neurons is imparted by other chemosensory inputs and not due to any intrinsic pH sensitivity of RTN neurons themselves (Gourine et al., 2010; Wu et al., 2019). Viewed in this context, the RTN serves as a relay, with GPR4 and TASK-2 functioning simply to maintain neuronal excitability rather than as sensors required for RTN activation by increases in CO_2_/H^+^.

In this study, we use CRISPR/Cas9 to generate multiple lines of knock-in mice expressing different variants of pH-desensitized GPR4 (Liu et al., 2010) to determine if pH sensitivity of GPR4, per se, is required for a normal HCVR and for activation of RTN neurons by increased CO_2_/H^+^. We find that CO_2_-stimulated breathing and CO_2_/H^+^ activation of RTN neurons are indeed blunted in these mice, indicating that intrinsic pH sensitivity of GPR4 is critical for respiratory chemoreception.

## Materials and Methods

### Animals

Experiments were performed on mice of either sex following procedures adhering to National Institutes of Health Animal Care and Use Guidelines and approved by the Animal Care and Use Committee of the University of Virginia (Protocol Number 2454). Mice were housed in HEPA-ventilated racks and steam-sterilized caging (up to five per cage), with *ad libitum* access to food and water. Animals were exposed to 12 h light/dark cycles in a vivarium maintained at 22−24°C and ∼40–50% relative humidity. We used multiple mouse lines for these experiments, including two novel GPR4 point mutant lines (GPR4-H81F, GPR4-H167F) that were generated using CRISPR-assisted genome editing technology. For this, single-guide RNAs were selected based on a search via the CRISPR guide design algorithm CRISPOR (http://crispor.tefor.net/), and the H81F (CAC > TTT) and H167F (CAC > TTC) point mutations were introduced into a 200 mer single-stranded oligodeoxynucleotide (ssODN) repair template developed from the *Gpr4* gene sequence, along with a PAM site mutation and translationally silent restriction site mutations. All reagents (crRNA, tracrRNA, Cas9, and ssODNs) were purchased from Integrated DNA Technologies. The Genetically Engineered Murine Model Core (University of Virginia) performed the gene targeting essentially as described (Gonye et al., 2024). In brief, CRISPR reagents were electroporated into fertilized eggs obtained from B6SJLF1/J females (GPR4-H81F) or C57BL/6J females (GPR4-H167F) and implanted into pseudopregnant foster mothers of ICR strain (Envigo). Pups born to the foster mothers were screened using tail snip DNA by PCR genotyping, diagnostic restriction digest, followed by Sanger's sequencing; germline transmission of the desired alleles was confirmed by breeding the founders with wild-type (WT) C57BL/6 mice (The Jackson Laboratory). The F1 heterozygotes were intercrossed to yield littermates that were homozygous for the WT or modified GPR4 alleles. For some experiments, the GPR4-H81F mice were further crossed to a Phox2b-green fluorescent protein (GFP) BAC transgenic mouse line (Jx99) in which GFP expression is driven by the Phox2b promoter; Jx99 mice were developed by the GENSAT project on an FVB/N × CD1 background, maintained in-house, and characterized previously (Lazarenko et al., 2009). This yielded homozygous control (Jx99) and GPR4-mutated (H81F-Jx99) littermates in which RTN neurons could be targeted for electrophysiological recording or for use in single-cell quantitative RT-PCR. Finally, a further cross of these H81F-Jx99 mice was made onto a previously described TASK-2 knock-out line originally generated using an exon trapping approach and backcrossed onto the C57BL6/J genetic background for 10 generations before intercrossing the progeny (Leighton et al., 2001; Gestreau et al., 2010; Wang et al., 2013). This yielded four additional genotypes of mixed background homozygous littermates for experimental study: GPR4^H81/H81^ (HH) or GPR4^H81F/H81F^ (FF) that were either TASK-2^+/+^ (++) or TASK-2^−/−^ (−).

In total, 279 mice were used for the data presented in this work (H81F line, *N* = male: HH, 13; FF, 21; *N* = female: HH, 15; FF, 10; H81F-Jx line, *N* = male: HH, 28; FF, 22; *N* = female: HH, 27; FF, 34; H167F line, *N* = male: HH, 14; FF, 12; *N* = female: HH, 11; FF, 13; H81F-TASK-2 line, *N* = male: HH/++, 5; FF/++, 10; HH/−, 7; FF/−, 5; *N* = female: HH/++, 9; FF/++, 6; HH/−, 6; FF/−, 11). Mice were randomly assigned to experimental groups, and all studies were performed and analyzed by individuals blinded to genotype and experimental treatment.

### Glosensor cAMP assay

HEK293T cells were plated in a poly-L-lysine-coated white 96-well plate (Greiner Bio-One 655074) at a density of 5 × 10^4^ cells per well in high-glucose DMEM (Invitrogen 11965-092) with sodium pyruvate and 10% fetal bovine serum. Cells were allowed to incubate overnight at 37°C/5% CO_2_. The following day, cells were transfected with the GloSensor -22F cAMP plasmid (Promega E2301) and WT or mutant GPR4 (final concentration 0.02 ng/µl); all GPR4 constructs contain a C-terminal myc-tagged mouse GPR4 expressed in pcDNA3.1 (Kumar et al., 2015). Constructs were mixed with Lipofectamine 2000 (Thermo Fisher Scientific 11668027) and added to cells according to manufacturer instructions and allowed to incubate for 20 h. The next day, transfection medium was removed and replaced with Hank's balanced salt solution (HBSS; Invitrogen 14175-095) containing 2% *v*/*v* GloSensor Reagent (Promega E1290). Cells were equilibrated for 2 h at 37°C/5% CO_2_. After equilibration, the solution was replaced with HBSS or containing 10 µM forskolin (Sigma-Aldrich F3917) as a positive control and incubated for 20 min at room temperature. Luminescence was detected using a Synergy HTX multimode plate reader.

### Cell surface biotinylation and Western blot

HEK293T cells were grown until 80–90% confluent in poly-L-lysine-coated 10 cm dishes. The day after plating, cells were transfected with WT or histidine mutant mouse GPR4 and Lipofectamine 2000 (Thermo Fisher Scientific 11668027) according to manufacturer's instructions. Approximately 20 h after transfection, cells were processed for a cell surface biotinylation and streptavidin pulldown. Briefly, cells were incubated with 1.1 mg/ml EZ-Link Sulfo-NHS-LC-Biotin (Thermo Fisher Scientific 21335) in Dulbecco’s PBS (DPBS; Invitrogen 14190-144) at 4°C. Biotinylation was quenched with 100 mM glycine in DPBS. After DPBS washes, cells were lysed in 1× RIPA (EMD Millipore 20–188)/2% SDS containing protease inhibitor cocktail (Sigma-Aldrich P8340), 10 mM NaF, and 10 mM NaVO_3_ using a probe sonicator. Protein concentration was measured using the Bradford assay. For each pulldown, Strep-Tactin Superflow Plus (Qiagen) beads were added to 1 mg total protein in lysis buffer and incubated with rocking at room temperature for 1 h. Pulldowns and total protein samples were then incubated in the 1× Laemmli buffer (62.5% glycerol, 12.5% SDS, 0.5% bromophenol blue, 25% fresh 2-mercaptoenthanol in 30 mM Tris–HCl), pH 6.8, for 30 min at 37°C before running SDS-PAGE. After separation, protein was transferred to 0.45 µm nitrocellulose membrane and blocked with 5% dry milk in TBST (10 mM Tris, 150 mM NaCl, and 0.1% Tween 20), pH 7.4. After blocking, membranes were incubated in primary antibody overnight at 4°C. Amersham ECL horseradish peroxidase (HRP)-linked secondary antibodies (GE HealthCare; anti-rabbit IgG, NA9340V, or anti-mouse IgG, NA931V; 1:10,000) and Western Lightning Plus ECL were used to visualize immunoreactive signals on Amersham Hyperfilm ECL (GE HealthCare).

### Immunocytochemistry

HEK293T cells were plated onto poly-L-lysine-coated 12 mm glass coverslips in a 24-well plate. Cells were transfected with WT or histidine mutant mouse GPR4 and Lipofectamine 2000 (Thermo Fisher Scientific) according to manufacturer's instructions. Approximately 20 h after transfection, cell culture medium was removed, and cells were washed with DPBS and permeabilized and blocked in PBS containing 10% fetal horse serum (FHS) and 0.3% Triton X-100. After blocking, coverslips were incubated in primary antibody solution (PBS/0.3% Triton X-100/1% FHS/1% bovine serum albumin) overnight at 4°C. Coverslips were washed with PBS/1% BSA and incubated in secondary antibody solution for 1 h at room temperature in the dark. DAPI was added during the last minute of secondary antibody incubation to label nuclei. Coverslips were mounted using ProLong Gold antifade reagent with DAPI (Invitrogen P36935) before imaging on a Zeiss LSM 700 scanning confocal microscope.

### Whole-body plethysmography

Ventilatory responses were measured in conscious, freely moving mice by whole-body plethysmography in chambers manufactured by Data Sciences International and recorded with the IOX software (emka TECHNOLOGIES). A mass flow regulator provided quiet, constant, and smooth flow through the animal chamber (0.5 L/min). Mice were familiarized with the plethysmography chamber the day prior to testing (3–4 h acclimation period) and again immediately before the testing protocol (for at least 2 h). The typical protocol entailed three sequential incrementing CO_2_ challenges (7 min exposures to 2, 4, 6, 8% CO_2_, balance O_2_; each separated by 5 min of 100% O_2_). Hypercapnic exposure was performed in hyperoxia to minimize contributions of peripheral chemoreceptors to the HCVR and attribute ventilatory effects to central chemoreception (Pepper et al., 1995). CO_2_ tension in the chambers was verified with a capnograph. Animals were also exposed to normoxic (21% O_2_, balance N_2_) and hypoxic (10% O_2_, balance N_2_) gas mixtures. After data collection, Poincaré analysis of the breathing frequency over the final 3 min of each challenge period (CO_2_, normoxia, or hypoxia) was performed to select periods of regular, calm breathing for analysis. For c-Fos-based analysis of CO_2_-activated neurons in vivo based on Fos expression, we habituated adult mice (60–350 d old) to the plethysmography chamber for 4–6 h on the day before the experiment and again for 2 h prior to the protocol. Mice were then exposed to the CO_2_ stimulus (12% CO_2_/60% O_2_/28% N_2_) for 45 min. For RNAscope detection of Fos, mice were anesthetized, examined for the absence of response to a firm toe pinch, and perfused transcardially with fixative immediately following the CO_2_ exposure; for c-Fos protein detection, CO_2_ exposure was followed by 45 min of hyperoxia before perfusion.

### Immunohistochemisty

Mice were anesthetized with ketamine/xylazine (200 mg/kg and 14 mg/kg, i.p.), examined for the absence of response to a firm toe pinch, perfusion-fixed (4%PFA/0.1 M PB), and tissue sections (30 µm, 1:3 serial) were prepared as previously described (Fortuna et al., 2009). Sections were stored at −20°C in cryoprotectant solution consisting of the following: 0.05 M sodium phosphate buffer (PB), 30% ethylene glycol, and 20% glycerol. All primary and secondary antibodies used in this study are listed in Table 1. Upon removal from the cryoprotectant solution, sections were washed in 0.1 M PB and then Tris saline (TS, 0.1 M Tris, 0.15 M NaCl). Sections were blocked in TS containing 0.3% Triton X-100 and 10% FHS at room temperature and incubated in primary antibody solution (TS/0.1% Triton X-100/1% FHS) overnight at 4°C with gentle rocking. Sections were washed in TS before incubation for 90 min at room temperature in secondary antibody solution (TS). DAPI solution was added during the last minute of secondary antibody incubation period. Sections were mounted on SuperFrost Plus glass slides (Thermo Fisher Scientific 12-550-15) sealed with ProLong Gold antifade reagent with DAPI (Invitrogen P36935) before imaging on a Zeiss Axioimager Z1 widefield epifluorescence microscope.

**Table 1.** Antibodies used in this study

Antibody	Product Number	RRID	Application(s)	Dilution
Mouse anti-myc	Cell Signaling Technology 2276S	AB_331783	WB/ICC	1:1,000/1:4,000
Mouse anti-tubulin	Sigma-Aldrich T9026	AB_477593	WB	1:8,000
Rabbit anti-CD46	LSBio LS-C331615	AB_2940833	WB/ICC	1:1,000/1:75
Mouse anti-TPH	Sigma-Aldrich T0678	AB_261587	IHC	1:250
Sheep anti-mouse HRP	GE HealthCare NA9310V	AB_772193	WB	1:10,000
Donkey anti-rabbit HRP	GE HealthCare NA9340V	AB_772191	WB	1:10,000
Donkey anti-mouse Alexa Fluor 488	Jackson ImmunoResearch Laboratories 715-546-150	AB_2340849	ICC	1:500
Donkey anti-rabbit Cy3	Jackson ImmunoResearch Laboratories 711-166-152	AB_2313568	ICC	1:500
Chicken anti-GFP	Aves Labs GFP-1010	AB_2307313	IHC	1:1,000
Goat anti-c-Fos	Santa Cruz Biotechnology sc-52-G	AB_2629503	IHC	1:1,000
Donkey anti-chicken Alexa Fluor 488	Jackson ImmunoResearch Laboratories 703-546-155	AB_2340376	IHC	1:500
Donkey anti-goat Cy3	Jackson ImmunoResearch Laboratories 705-166-147	AB_2340413	IHC	1:500

WB = western blot; ICC = immunocytochemistry; IHC = immunohistochemistry.

**Table 2.** Arterial blood gas analysis of GPR4(HF) mutant mouse lines

Genotype	WT (12)	H81F (13)	Jx99 (13)	H81F-Jx (13)	WT (15)	H167F (14)
pH	7.40 ± 0.01	7.44 ± 0.02*	7.44 ± 0.01	7.44 ± 0.02	7.42 ± 0.01	7.42 ± 0.02
PCO_2_ (mmHg)	30.4 ± 1.2	34.3 ± 1.5	30.8 ± 0.9	29.9 ± 2.2	33.5 ± 1.0	31.2 ± 1.0
$\mathrm{HC}O_{3}^{-}$ (mM)	19.8 ± 0.9	24.3 ± 0.9**	21.2 ± 0.9	20.3 ± 1.5	21.7 ± 0.8	20.2 ± 0.9
PO_2_ (mmHg)	84.3 ± 3.3	89.9 ± 2.3	87.0 ± 2.5	89.9 ± 4.1	85.8 ± 2.2	91.2 ± 2.3
lactate (mM)	3.7 ± 0.7	3.4 ± 0.3	2.7 ± 0.4	3.7 ± 0.4	4.0 ± 0.5	4.0 ± 0.5

Statistical analysis by unpaired *t* test between WT and HF mutant littermates (*N*) for each line.

* *p* = 0.0275.

** *p* = 0.0023.

### RNAscope in situ hybridization

*Nmb*, *Gpr4*, *Kcnk5*, and *Fos* transcripts were detected using the RNAscope platform (Advanced Cell Diagnostics, ACD). Following tissue fixation and sectioning, tissue sections were mounted on SuperFrost Plus slides and allowed to air-dry overnight. Sections were washed twice in sterile water before a 30 min incubation in RNAscope Protease IV solution (ACD 322336) at 40°C. After protease treatment, slides were washed in sterile water and then incubated with probes for mouse *Nmb* (ACD 459931-C2), *Gpr4* (ACD 427941), *Kcnk5* (ACD 427951-C3), and/or *Fos* (ACD 316921-C3) for 2 h at 40°C. Following probe incubation, sections were processed according to manufacturer instructions for the Fluorescent Multiplex Detection Reagent Kit v1 (ACD 320851). After processing, sections were allowed to dry before slides were sealed with ProLong Gold antifade reagent with DAPI (Invitrogen P36935) before imaging on a Zeiss Axioimager Z1 widefield epifluorescence microscope.

### Cell counts and analysis

For analysis of all histochemical experiments, images of serial sections (1:3 series) through the rostrocaudal extent of the RTN and in brainstem raphe nuclei [raphe magnus and pallidus, RPa; raphe obscurus; parapyramidal (PPy) region] were acquired using an epifluorescence microscope (Zeiss AxioImager Z1) equipped with the Neurolucida software. Labeled cells were counted and aligned for averaging according to defined anatomical landmarks (Paxinos et al., 2001). Tracings were exported to the NeuroExplorer software (MBF Biosciences) for analysis of RTN and raphe cell number within the ventral brainstem. The text and figures present the actual number of cells counted from the 1:3 series of tissue sections, with no stereological correction factor applied (i.e., the actual number of cells would be ∼3 times higher).

### Acute slice preparation

For single-neuron collection and patch-clamp recordings from Jx99 WT and H81F-Jx mice, transverse brainstem slices were prepared as previously described (Shi et al., 2016, 2021). For neonates (P7–P13), animals were anaesthetized with ketamine and xylazine (375 and 25 mg/kg, i.p.); after establishing no response to firm toe pinch, the mice were rapidly decapitated, and brainstems were immediately removed and sliced in the coronal plane (300 µm) using a vibrating microslicer (DTK Zero 1; Ted Pella) in ice-cold, sucrose-substituted Ringer’s solution containing the following (in mM): 260 sucrose, 3 KCl, 5 MgCl_2_, 1 CaCl_2_, 1.25 NaH_2_PO_4_, 26 NaHCO_3_, 10 glucose, and 1 kynurenic acid. Slices were held in normal Ringer's solution containing the following (in mM): 130 NaCl, 3 KCl, 2 MgCl_2_, 2 CaCl_2_, 1.25 NaH_2_PO_4_, 26 NaHCO_3_, and 10 glucose. Cutting and holding solutions were constantly bubbled with 5% CO_2_/95% O_2_. Adult animals (P49–P109) were deeply anaesthetized by intraperitoneal injection of ketamine/xylazine (as above) and perfused transcardially with 25 ml of ice-cold NMDG-aCSF (in mM: 93 NMDG, 2.5 KCl, 1.2 NaH_2_PO_4_, 30 NaHCO_3_, 20 HEPES, 25 glucose, 5 Na-ascorbate, 2 thiourea, 3 Na-pyruvate, 12 N-acetyl-L-cysteine, 10 MgSO_4_, 0.5 CaCl_2_, with 10N HCl), pH adjusted to 7.3–7.4. Animals were rapidly decapitated, and heads were submerged in NMDG-aCSF. Brainstems were removed and sliced in the coronal plane (150 µm) with a vibrating microslicer in NMDG-aCSF. After a brief recovery period (≤12 min at 32–34°C) in NMDG-aCSF, slices were held in HEPES-aCSF (in mM: 92 NaCl, 2.5 KCl, 1.2 NaH_2_PO_4_, 30 NaHCO_3_, 20 HEPES, 25 glucose, 5 Na-ascorbate, 2 thiourea, 3 Na-pyruvate, 12 N-acetyl-l-cysteine, 2 MgSO_4_, 2 CaCl_2_, with KOH or HCl if necessary), pH was adjusted to 7.3–7.4, until use. All solutions were constantly bubbled with 5% CO_2_/95% O_2_.

### Single-cell RT-qPCR

Individual GFP-labeled RTN neurons were harvested under direct vision from mouse brainstem slices (*n* = 176 cells; *N* = 25 mice) in a HEPES-based solution containing the following (in mM): 140 NaCl, 3 KCl, 2 MgCl_2_, 2 CaCl_2_, 10 HEPES, 10 glucose, pH 7.4 at room temperature, in the recording chamber of a fluorescence microscope (Zeiss Axioimager FS, Carl Zeiss Microscopy). Neurons were targeted based on a healthy appearance (e.g., soma size and turbidity, membrane transparency, dendritic process visibility) and fluorescence intensity. A pipette loaded with a sterile HEPES-based buffer (tip diameter, ∼10 µm) was advanced toward the cell, with application of gentle positive pressure to clear away nearby cellular debris and extracellular matrix (delivered by mouth, via a side port on the pipette holder with an intervening 0.22 µm sterile filter in the line). Subsequently, gentle suction was used to collect the cell while minimizing aspiration of nonsomatic cellular components. Once the cell was picked, ∼1 µl of internal solution containing the cytoplasmic contents was expelled into a sterile tube containing reverse transcriptase reaction reagents (SuperScript III First-Strand, Invitrogen 18080-051). Neurons were analyzed simultaneously for expression of multiple transcripts (*Nmb*, *Slc17a6*, *Gpr4*, *Kcnk5*, *Gapdh*) by single-cell multiplex quantitative RT-PCR (sc-qPCR). We used primer sets for sc-qPCR that yielded short amplicons [see Shi et al. (2016, 2021) for primer sequences]; the cycle threshold (Ct) levels of test transcripts were rescaled by their average, transformed into relative quantities using the amplification efficiency, normalized to *Gapdh* [an internal reference gene; ΔCt = Ct(test) – Ct(*Gapdh*)], and expressed as 2^−Δ*C*t^ (Pfaffl, 2001).

### In vitro neuronal electrophysiology

Cell-attached and whole-cell recordings of pH sensitivity of GFP-labeled RTN neurons were performed in transverse brain slices (300 µm) prepared from neonatal WT Jx99 or H81F-Jx animals (P7–P13), as described above. Slices were placed in a chamber on a fixed-stage fluorescence microscope equipped with fluorescence and infrared optics (Zeiss AxioSkop) at room temperature in HEPES-based buffer containing the following (in mM): 140 NaCl, 3 KCl, 2 MgCl_2_, 2 CaCl_2_, 10 HEPES, 10 glucose, with pH adjusted between 7.0 and 7.8 by addition of HCl or NaOH. Patch electrodes (3–6 MΩ) were filled with the following (in mM): 120 KCH_3_SO_3_, 4 NaCl, 1 MgCl_2_, 0.5 CaCl_2_, 10 HEPES, 10 EGTA, 3 Mg-ATP, and 0.3 GTP-Tris, pH 7.2, adjusted with KOH. Firing activity was recorded using pCLAMP software, an Axoclamp 200B amplifier, and a Digidata 1322A digitizer (Molecular Devices). All recordings were made in the presence of strychnine (30 µM), bicuculline (10 µM), and 6-cyano-7-nitroquinoxaline-2,3-dione (10 µM). For cell-attached recordings, cells were held at −60 mV under voltage clamp (Perkins, 2006). Firing rate histograms of RTN neuronal discharge were generated by integrating action potential discharge in 10 s bins using the Spike2 software (Cambridge Electronic Design), and the pH sensitivity of individual RTN neurons was assessed by linear regression analysis to obtain a pH 50 value, that is, the pH at which firing rate was half that obtained at pH 7.0 (Wang et al., 2013; Kumar et al., 2015). For whole-cell current–clamp recordings, cells were held at −60 mV via DC current injection before challenges with current step protocols.

### Blood gas analysis

Mice were habituated to a tail warmer and restraint apparatus (Braintree Scientific) on two occasions before blood was sampled. On the day of sampling, mice were habituated to the laboratory space for ∼2 h following transportation from the vivarium and then gently restrained for at least 30 min before blood sampling. Arterial blood from the ventral tail artery (∼100 µl) was collected from the awake mouse into a heparinized capillary tube and immediately analyzed with an iSTAT hand held analyzer (CG4+ cartridge, Heska).

### Statistics

All statistical analyses were performed using GraphPad Prism (v. 10.2); details of specific tests are provided in the text or figure legends, and parametric tests were used when data were normally distributed (Shapiro–Wilk test). Data are presented in box and whiskers format (the median bisects a box bounded by the 25th percentile and 75th percentile, with whiskers depicting the range), or as mean ± standard error of the mean (SEM). Statistical significance was set at *p* < 0.05.

## Results

### Histidine mutations disrupt pH sensitivity of mouse GPR4

**Figure 1.**
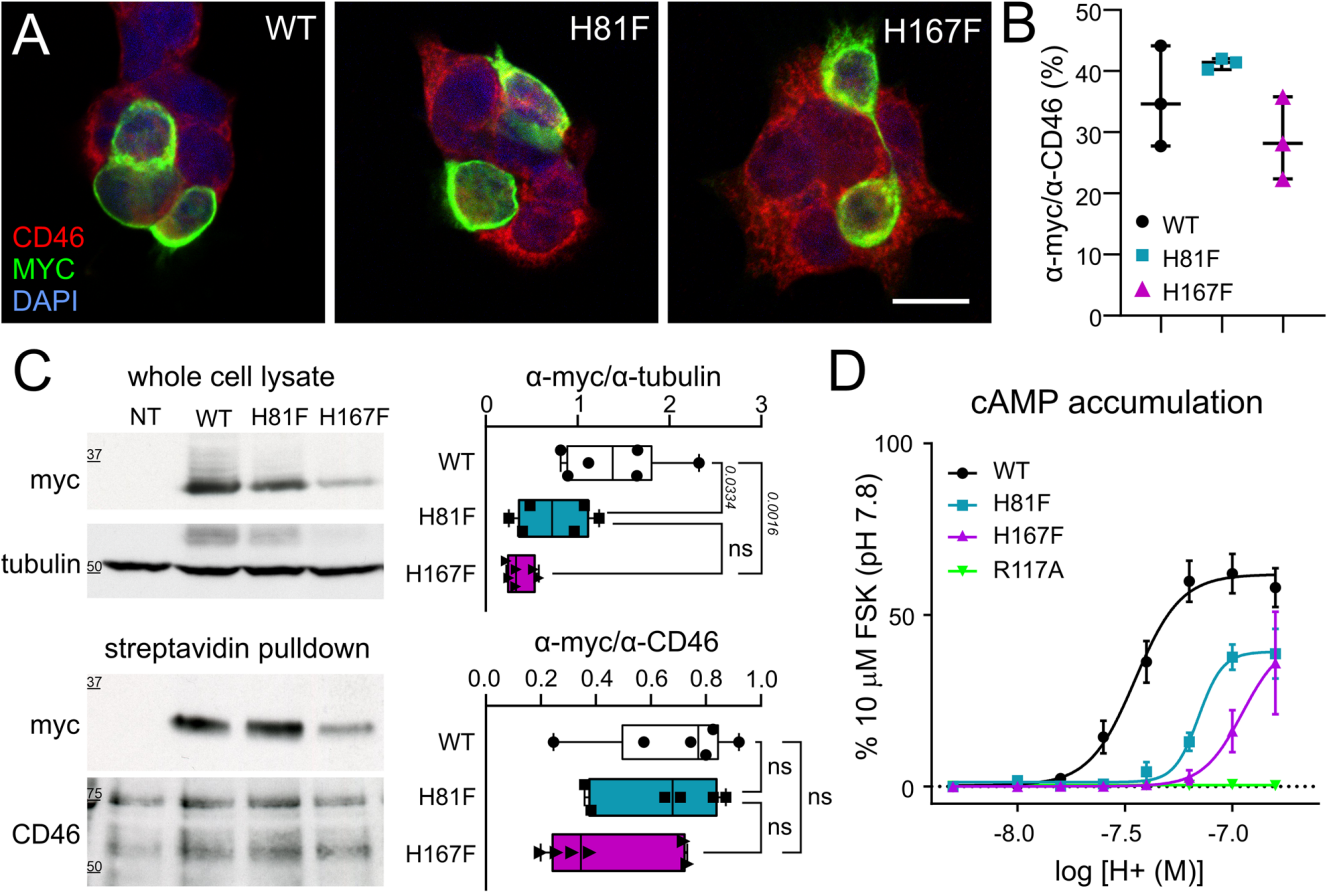
His-mutated GPR4 constructs expressed on the cell surface of HEK293T cells show blunted pH-dependent cAMP accumulation. ***A***, Representative images of WT and histidine mutant GPR4-myc constructs expressed in HEK293T cells; scale bar, 30 µm. ***B***, Average transfection efficiency (% myc-positive cells) of WT and histidine mutant mGPR4-myc constructs. ***C***, Left, Representative Western blot showing whole-cell (top) and cell surface (bottom) expression of the indicated myc-tagged GPR4 constructs expressed in HEK293T cells. Right, Aggregate data from six independent experiments. *P* values displayed are from one-way ANOVA with Tukey's multiple-comparison test (whole cell, *F*_(2,15)_ = 9.628; *p* = 0.002; cell surface, *F*_(2,15)_ = 1.968; *p* = 0.1742). GPR4 protein levels are normalized to control cytosolic (tubulin) and cell membrane (CD46) protein markers. ***D***, GloSensor luminescence assay of pH-dependent cAMP accumulation in HEK293T cells transfected with mGPR4-myc constructs: WT (*n* = 5), H81F (*n* = 3), H167F (*n* = 3), or Gα binding-deficient (R117A, *n* = 3). *n* represents a biological replicate (independent transfections).

GPR4 was identified as a proton-activated and adenylyl cyclase-stimulating receptor containing an extracellular shell of titratable histidine residues (Ludwig et al., 2003); subsequent mutational analyses demonstrated that three specific histidine residues in human GPR4 (corresponding to His81, His167, and His271 in the mouse receptor) are required for pH-sensitive GPR4 signaling without affecting receptor expression (Liu et al., 2010). Similarly, we found that mouse GPR4 constructs containing either of two histidine to phenylalanine substitutions—GPR4(H81F) and GPR4(H167F)—could be expressed in HEK293T cells (Fig. 1*A*,*B*); although cytosolic expression levels of GPR4(H167F) appeared to be lower after transfection in this system, cell surface biotinylation and streptavidin pulldown assays revealed that both Phe-substituted mutants were present on the cell membrane at comparable levels to the WT receptor (Fig. 1*C*). Unlike the GPR4(R117A) variant that cannot signal to downstream effectors (Kumar et al., 2015), both GPR4(H81F) and GPR4(H167F) could transduce changes in extracellular acidification into elevated intracellular cAMP levels; however, they displayed a decreased sensitivity for proton activation, right-shifted pH 50, and a lower maximum level of cAMP accumulation, in comparison with WT GPR4 (Fig. 1*D*). The differences were pronounced at arterial pH levels expected during in vivo CO_2_ challenges, e.g., pH 7.6–7.2; (Guyenet et al., 2005), although some adenylyl cyclase activity became apparent near pH 7.2 for GPR4(H81F).

### CO_2_-stimulated breathing is blunted in GPR4(H81F) knock-in mice

**Figure 2.**
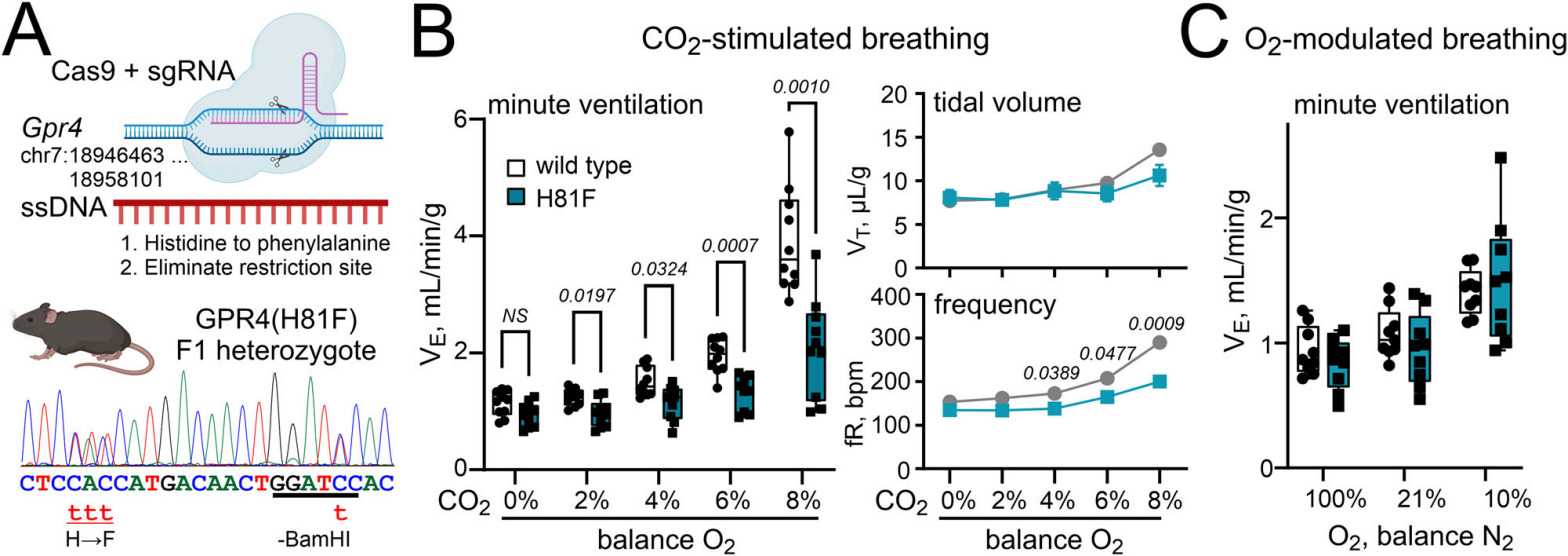
Mutation of a pH-sensing residue (His81) in GPR4 blunts CO_2_-stimulated breathing in mice without affecting oxygen-modulated breathing. ***A***, Top, Schematic of CRISPR knock-in strategy. Bottom, Sanger sequencing trace from an F1 heterozygote showing targeted alterations in *Gpr4* genomic sequence: substitution of Phe for His81 and elimination of BamHI restriction site. ***B***, Minute ventilation, tidal volume, and frequency of GPR4(H81F) and WT control mice (*N* = 9 each, P52–P87) in response to increasing levels of inspired CO_2_ (balance O_2_). Two-way repeated–measure (RM) ANOVA: minute ventilation, *F*_(1,18) _= 26.75; *p* < 0.0001 for genotype and *F*_(4,72) _= 15.04; *p* < 0.0001 for CO_2_ exposure × genotype; frequency, *F*_(1,18) _= 17.65; *p* = 0.0005 for genotype and *F*_(4,72) _= 6.311; *p* = 0.0002 for CO_2_ exposure × genotype; tidal volume, *F*_(1,18) _= 0.7610; *p* = 0.3945 for genotype and *F*_(4,72) _= 7.138; *p* < 0.0001 for CO_2_ exposure × genotype. *P* values displayed are from two-way RM ANOVA analysis with Šidák's multiple-comparison test. ***C***, Minute ventilation of H81F and WT control mice (*N* = 9 each) during exposure to the indicated levels of inspired O_2_. Two-way RM ANOVA for minute ventilation: *F*_(1,16) _= 0.1569; *p* = 0.6973 for genotype and *F*_(2,32) _= 0.1231; *p* = 0.1231 for O_2_ exposure × genotype.

We previously showed that global genetic deletion of GPR4 in mice decreased CO_2_/H^+^ sensitivity of RTN respiratory chemosensory neurons and strongly reduced CO_2_-stimulated breathing (Kumar et al., 2015). To examine the role of GPR4 pH sensitivity, per se, we leveraged CRISPR/Cas9 genome editing to generate global knock-in mice expressing the pH-desensitized GPR4(H81F) receptor from the endogenous *Gpr4* locus in mice (Fig. 2*A*) and intercrossed F1 heterozygous GPR4^H81F/+^ mice to produce WT GPR4^+/+^ and GPR4^H81F/H81F^ littermates on a mixed genetic background (B6SJLF1/J) for experimental study (hereafter called H81F).

We performed whole-body plethysmography to assess CO_2_-stimulated breathing in WT and GPR4(H81F) mice, examining a range of CO_2_ levels (0 to 8%; under hyperoxic conditions to minimize the influence of peripheral chemoreceptors). In comparison with WT littermates, H81F mice had significantly reduced ventilatory response to CO_2_ (Fig. 2*B*); the CO_2_-induced increase in minute ventilation (V_E_), the product of respiratory frequency (fR), and tidal volume (V_T_) was significantly blunted in H81F mice (ΔV_E_ in 8% CO_2_ reduced by ∼59%, from 2.8 ± 0.3 ml/min/g to 1.1 ± 0.2 ml/min/g; *N* = 10 each; *p* = 0.0002). These respiratory deficits were largely due to effects on fR (Fig. 2*B*). Moreover, they were specific to CO_2_ sensitivity as there was no difference in baseline respiration in room air (21% O_2_) between WT and H81F animals and also no difference in their hypoxic ventilatory response (10% O_2_; Fig. 2*C*). These results in GPR4(H81F) mice were similar to those obtained from global GPR4 knock-out mice (Kumar et al., 2015).

### CO_2_ sensitivity of RTN neurons is reduced in GPR4(H81F) knock-in mice

**Figure 3.**
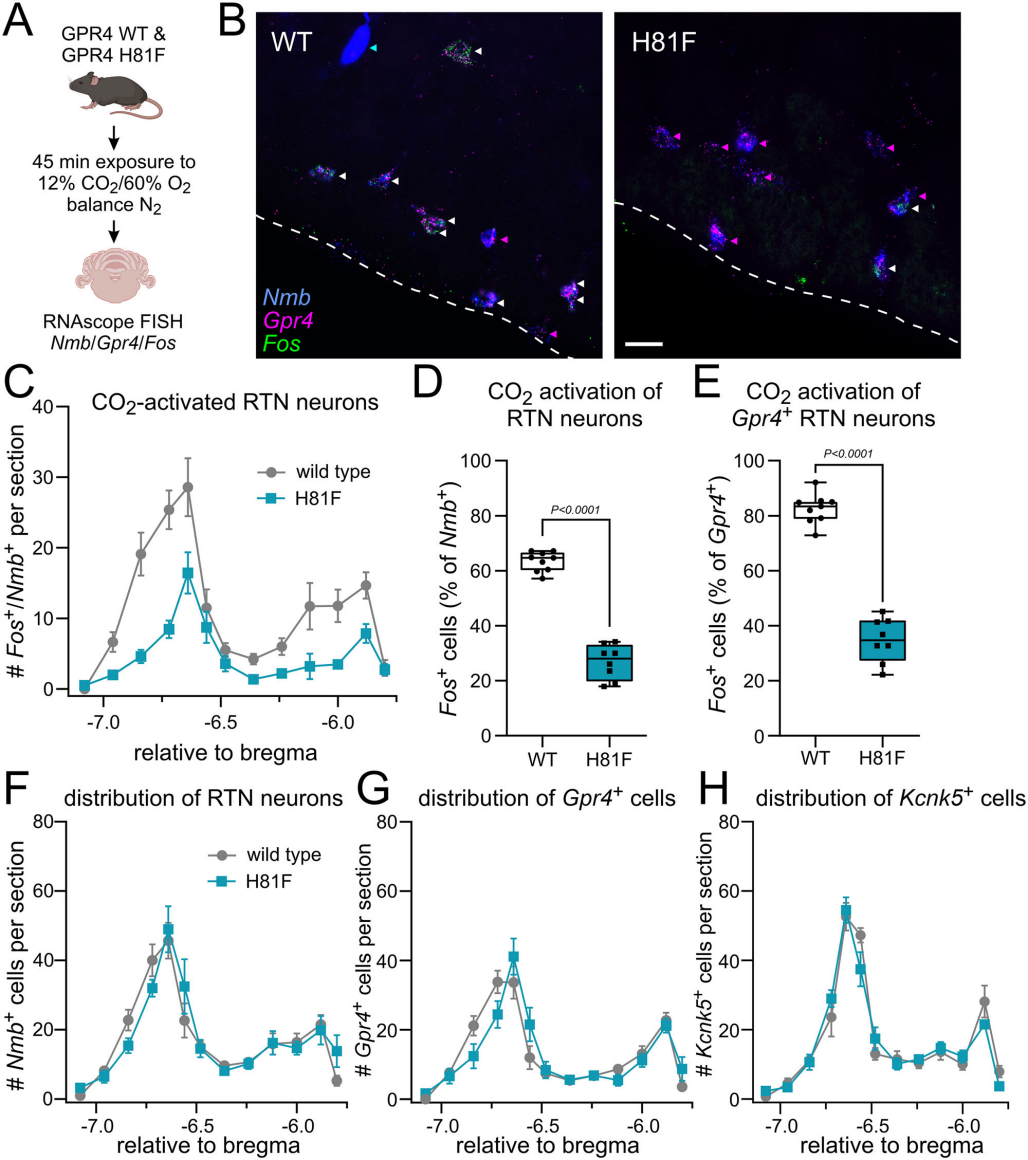
CO_2_-stimulated RTN neuron activation in vivo is blunted in GPR4(H81F) mice. ***A***, Schematic of experimental design. ***B***, Representative RNAscope images for *Nmb*, *Gpr4*, and *Fos*. CO_2_-activated GPR4–expressing RTN neurons (*Nmb*^+^/*Gpr4*^+^/*Fos*^+^) are denoted with white arrowheads; GPR4-expressing RTN neurons (*Nmb*^+^/*Gpr4*^+^) not activated by CO_2_ (i.e., *Fos*-negative) are denoted with magenta arrowheads. The blue arrowhead denotes an *Nmb*^+^ cell expressing neither *Gpr4* nor *Fos*. ***C–E***, Distribution of CO_2_-activated RTN neurons (*Fos*^+^/*Nmb*^+^) throughout the rostrocaudal extent of the RTN in WT and H81F mice (***C***, mean ± SEM; *N* = 9 and 8, P47–P334) and the total percentage of *Fos*-expressing *Nmb*^+^ (i.e., RTN) neurons (***D***) and *Gpr4*^+^ RTN neurons also positive for *Fos* (***E***). Unpaired *t* test. ***F–H***, Rostrocaudal distribution of RTN neurons (*Nmb*^+^, ***F***) and of *Gpr4*^+^-expressing (***G***) and *Kcnk5*^+^-expressing (***H***) subsets of RTN neurons in WT and H81F mice [mean ± SEM, (***F***, ***G***) *N* = 9 and 8; (***H***) *N* = 7 and 7; P47–P334].

We examined the role of GPR4-mediated pH sensitivity in mediating CO_2_/H^+^ activation of the respiratory chemosensory neurons of the RTN in vivo. First, using RNAscope in situ hybridization, we examined *Fos* expression after 12% CO_2_ exposure as a surrogate measure of CO_2_-mediated activation of *Gpr4*-expressing RTN neurons; RTN neurons were definitively identified by expression of *Nmb* (Fig. 3*A*; Shi et al., 2017). Consistent with reduced CO_2_ sensitivity, we found fewer *Fos*-labeled *Nmb*^+^/*Gpr4*^+^ RTN neurons (white arrowheads) in sections from H81F mice (Fig. 3*B*). Indeed, the number of CO_2_-activated RTN neurons (i.e., *Fos*^+^/*Nmb*^+^ cells) was lower in H81F mice relative to WT littermates across the rostrocaudal extent of the nucleus (Fig. 3*C*). We found that 63.4 ± 1.2% of RTN neurons in WT mice were activated by CO_2_, similar to our previous work (Shi et al., 2021; Li et al., 2023), whereas only 26.8 ± 2.2% of RTN neurons were activated in H81F mice (Fig. 3*D*). Note that a subpopulation of RTN neurons with high levels of *Nmb* do not express *Gpr4* (Fig. 3*B*, blue arrowhead) and those cells also tend not to express Fos after CO_2_ exposure (Shi et al., 2017); thus, when focusing only on the *Gpr4*-expressing subgroup of RTN neurons, we found a higher percentage of *Fos*^+^, CO_2_-activated neurons in WT mice (82.6 ± 1.8%) that was still greater than observed in H81F mice (34.8 ± 2.8%; Fig. 3*E*). Notably, we found no difference in the number or distribution of *Nmb*-expressing cells (i.e., RTN neurons) throughout the nucleus (Fig. 3*F*) or in the number/distribution of RTN neurons that express *Gpr4* or *Kcnk5* (Fig. 3*G*,*H*). These data indicate that effects of the H81F substitution in GPR4 on CO_2_-stimulated breathing are not due to differences in the number of RTN neurons, including the *Gpr4*- and *Kcnk5*-expressing populations; they also suggest that RTN neurons expressing GPR4(H81F) may be less sensitive to CO_2_.

### The GPR4(H81F) substitution reduces pH sensitivity of RTN neurons in vitro

**Figure 4.**
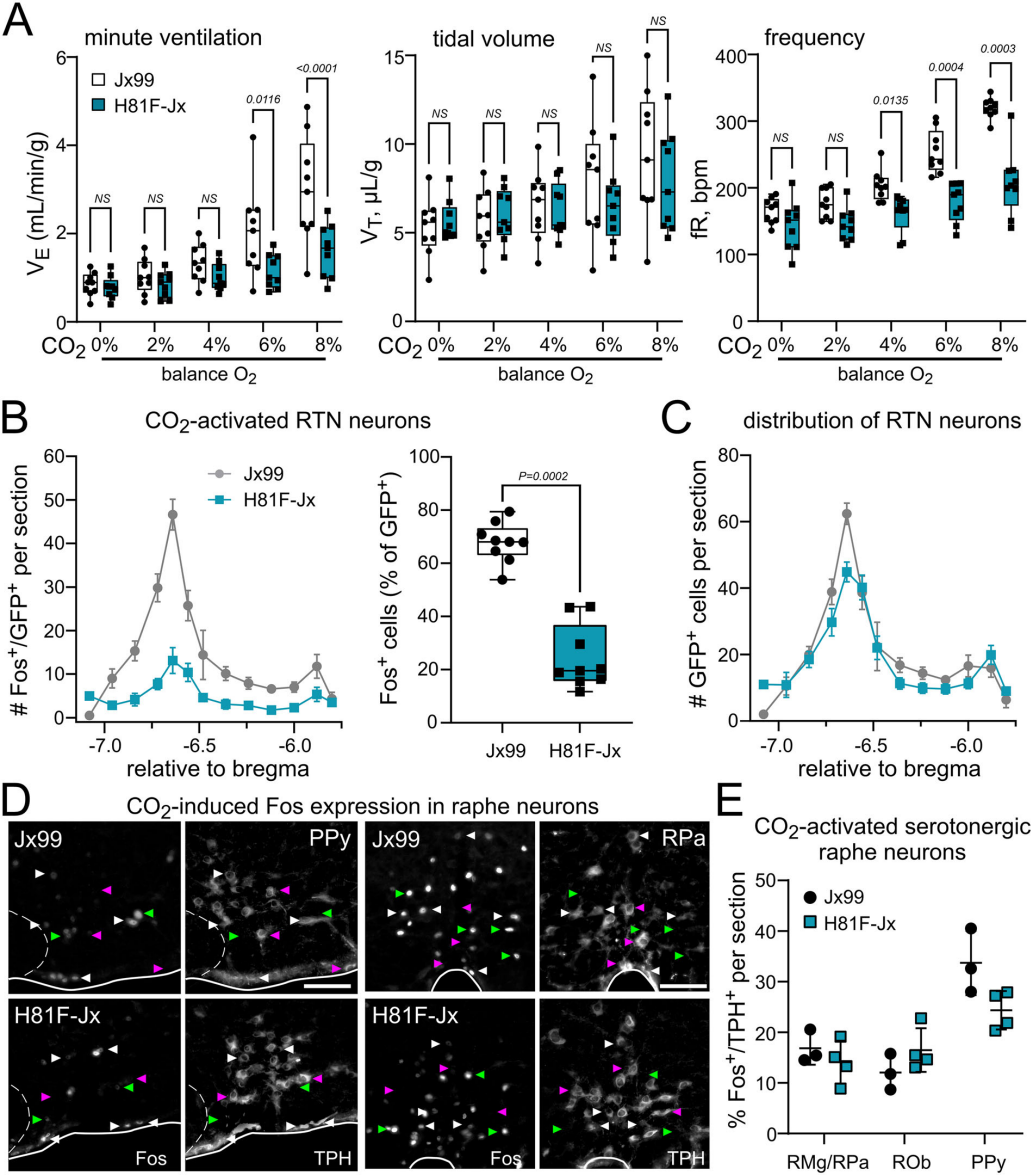
CO_2_-stimulated breathing and RTN neuron activation is decreased in GPR4(H81F) mice on a Phox2b-GFP (Jx99) background. ***A***, CO_2_-stimulated V_E_ (left), V_T_ (middle), and fR (right) in H81F-Jx99 mice compared with WT Jx99 mice (mean ± SEM, *N* = 9 and 9, P54–P165). Two-way RM ANOVA: minute ventilation, *F*_(1,16) _= 5.801; *p*= 0.0284 for genotype and *F*_(4,64) _= 9.152; *p* < 0.0001 for CO_2_ exposure × genotype; frequency, *F*_(1,16) _= 31.81; *p* < 0.0001 for genotype and *F*_(4,64) _= 12.77; *p* < 0.0001 for CO_2_ exposure × genotype; tidal volume, *F*_(1,16) _= 0.2631, *p* = 0.615 for genotype and *F*_(4,64) _= 2.259; *p* = 0.0724 for CO_2_ exposure × genotype. ***B***, Distribution of Fos-immunoreactive, GFP-labeled neurons and total number of GFP-expressing neurons (***C***) throughout the rostrocaudal extent of the RTN in either WT Jx99 or H81F-Jx99 mice after exposure in vivo to 12% CO_2_ (mean ± SEM, *N* = 9 and 9, P54–P241; unpaired *t* test). ***D***, Representative images of brainstem sections from CO_2_-exposed WT Jx99 or H81F-Jx99 mice immunostained for Fos and tryptophan hydroxylase (TPH) in the PPy raphe and RPa. Activated serotonergic raphe neurons (Fos+/TPH+) are denoted by white arrowheads, Fos-labeled neurons that are not stained for TPH by green arrowheads, and TPH + neurons that were not activated by CO_2_ (i.e., Fos-negative) by magenta arrowheads. ***E***, The percentage of serotonergic neurons activated by CO_2_ within the indicated raphe nuclei from WT Jx99 or H81F-Jx99 mice (*N* = 3 and 4, P63–P135; *F*_(1,5) _= 3.069; *p* = 0.1402 for genotype, by two-way ANOVA).

To provide a more direct test of the effects of CO_2_/H^+^ sensitivity of RTN neurons, we performed in vitro electrophysiological experiments in acute brain slices from WT and GPR4(H81F) mice. In order to visualize RTN neurons for recording, we crossed the H81F mice with the previously described Phox2b-GFP mice (Jx99), a line in which we typically find >90% of GFP^+^ neurons in the RTN region increase firing in response to bath acidification (Lazarenko et al., 2009; Wang et al., 2013; Kumar et al., 2015). We first verified that the differences observed for in vivo CO_2_ sensitivity were retained in the H81F-Jx99 line, in which the GPR4-H81F substitution is expressed on a different mixed genetic background. Indeed, CO_2_-stimulated breathing was blunted in H81F-Jx99 mice, in comparison with their WT Jx99 control littermates (Δ*V_E_* in 8% CO_2_ reduced by ∼61%, from 2.1 ± 0.4 ml/min/g to 0.8 ± 0.1 ml/min/g; *N* = 9 each; *p* = 0.0040; Fig. 4*A*), and the number of Fos-immunoreactive RTN neurons observed after CO_2_ exposure was reduced (from 67.8 ± 2.5% to 24.3 ± 4.0%; *N* = 9 each), with no difference in the number or distribution of GFP^+^ neurons (Fig. 4*B*,*C*). We also examined CO_2_-evoked Fos immunoreactivity in raphe neurons, which also express GPR4 (Kumar et al., 2015; Hosford et al., 2018; Gonye et al., 2024), and found no difference in the percentage of serotonergic (i.e., TPH^+^) neurons of medullary raphe nuclei in mice with WT or GPR4(H81F) alleles (Fig. 4*D*,*E*). Thus, the physiological effects in this separate line of H81F-Jx99 mice prepared for electrophysiological studies phenocopied those from the initial GPR4(H81F) line as well as the GPR4 knock-out mice reported earlier (Kumar et al., 2015).

**Figure 5.**
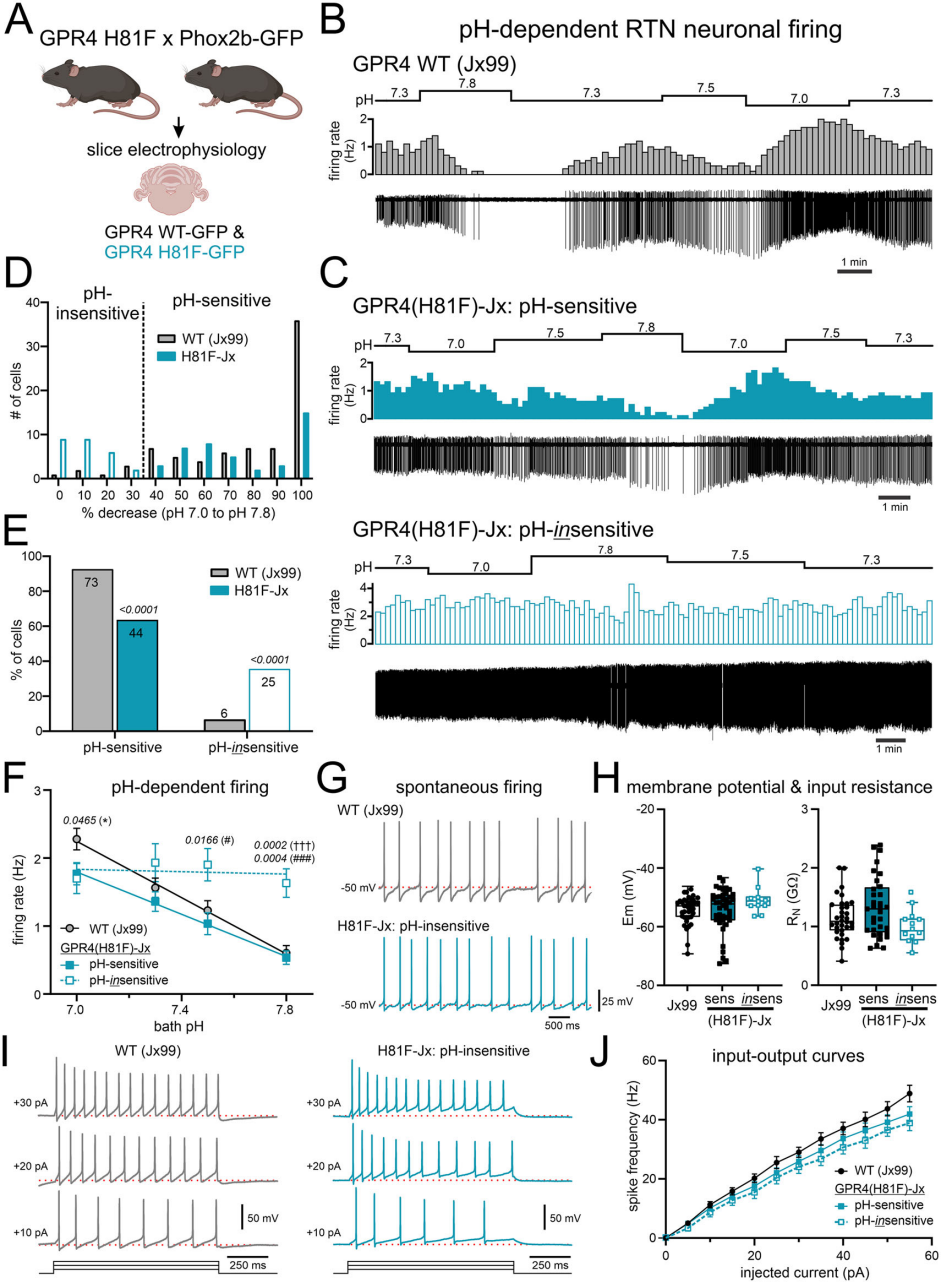
A subset of RTN neurons from GPR4(H81F) mice lack pH sensitivity in acute slices. ***A***, Schematic of experimental design to target GFP-labeled RTN neurons in slices from neonatal WT and H81F Phox2b::GFP (Jx99) mice (*N* = 40 and 41, P7–P13). ***B***, Representative cell-attached recording from a GFP-expressing RTN neuron from a Jx99 mouse. ***C***, Representative cell-attached recordings from pH-sensitive and pH-insensitive GFP^+^ RTN neurons from H81F-Jx99 mice. ***D***, Frequency distribution of pH-sensitive and pH-insensitive RTN neurons from WT Jx99 (*N* = 40) and H81F-Jx99 mice (*N* = 41); the cutoff for designation into these two groups is indicated. ***E***, The percentage of cells from each genotype that are either pH-sensitive or pH-insensitive (χ^2^ analysis; numbers of cells from each group are provided within the bars). ***F***, Averaged firing rates at different bath pH for RTN neurons from GPR4 WT Jx99 mice and for RTN neurons from H81F-Jx99 mice that were identified as either pH-sensitive or pH-insensitive (as in ***D***, ***E***). *compares Jx99 vs H81F-Jx99:pH-sensitive; †††compares Jx99 vs H81F-Jx99:pH-insensitive; #compares H81F-Jx99:pH-sensitive vs H81F-Jx99:pH-insensitive, by mixed-effect model with Tukey's test (for genotype *F*_(2,143) _= 2.553; *p* = 0.0814; for pH × genotype *F*_(6,410) _= 19.67; *p* < 0.0001). ***G***, Whole-cell current–clamp records from representative RTN neurons in slices from WT (Jx99) and H81F-Jx99:pH-insensitive mice showing spontaneous action potential firing at resting membrane potential (***G***, −50 mV). ***H***, Resting membrane potential (Em, *left*) and steady-state input resistance (R_N_, *right*) of RTN neurons from Jx99 mice and pH-sensitive and pH-insensitive RTN neurons from H81F-Jx99 mice. ***I***, Current-evoked firing during step current injections from a holding potential of −60 mV (indicated with dashed red line) in exemplar RTN neurons from WT (Jx99) and H81F-Jx99:pH-insensitive mice. ***J***, Firing frequency during current injection in RTN neurons from the indicated mice.

We performed cell-attached recordings from GFP-expressing RTN neurons to assess the effects of changing bath pH on cell firing (Fig. 5*A*). In slices from WT Jx99 mice in the presence of blockers of fast synaptic transmission, RTN neurons were spontaneously active at pH 7.3; they decreased their firing rate in response to bath alkalization to pH 7.5 and pH 7.8 and increased their firing rate in response to bath acidification to pH 7.0 (Fig. 5*B*). This characteristic response was observed in the majority of RTN neurons (>90%), for which a cell was considered pH-sensitive if the firing rate decreased by >30% between pH 7.0 and pH 7.8 (Fig. 5*D*,*E*). In contrast, we observed two types of responses in recordings of RTN neurons from H81F-Jx99 mice. For some cells (64%), bath alkalization and acidification led to decreased and increased firing, whereas a significant proportion (36%) of GPR4(H81F)-expressing RTN neurons did not display pH-modulated firing (Fig. 5*C*–*E*). The pH-sensitive neurons from H81F-Jx99 mice had a significantly lower firing frequency at pH 7.0 (Fig. 5*F*), and the pH-insensitive neurons maintained their firing across the pH range to present higher firing frequency at alkaline pH levels (Fig. 5*F*). We obtained whole-cell current–clamp recordings in a subset of RTN neurons. As exemplified for a WT Jx99 and a pH-insensitive H81F-Jx99 RTN neuron, cells fired spontaneously at pH 7.3, with an interspike membrane potential ∼−50 mV (Fig. 5*G*,*H*); from a −60 mV holding potential, RTN neurons discharged repetitively at increased frequency with depolarizing step current injections (Fig. 5*I*). We found no difference among any of the groups—i.e., WT Jx99 and H81F-Jx99 RTN neurons, pH-sensitive or pH-insensitive—in intrinsic properties (i.e., Em, *R_N_*; Fig. 5*H*) or input–output characteristics (Fig. 5*J*).

**Figure 6.**
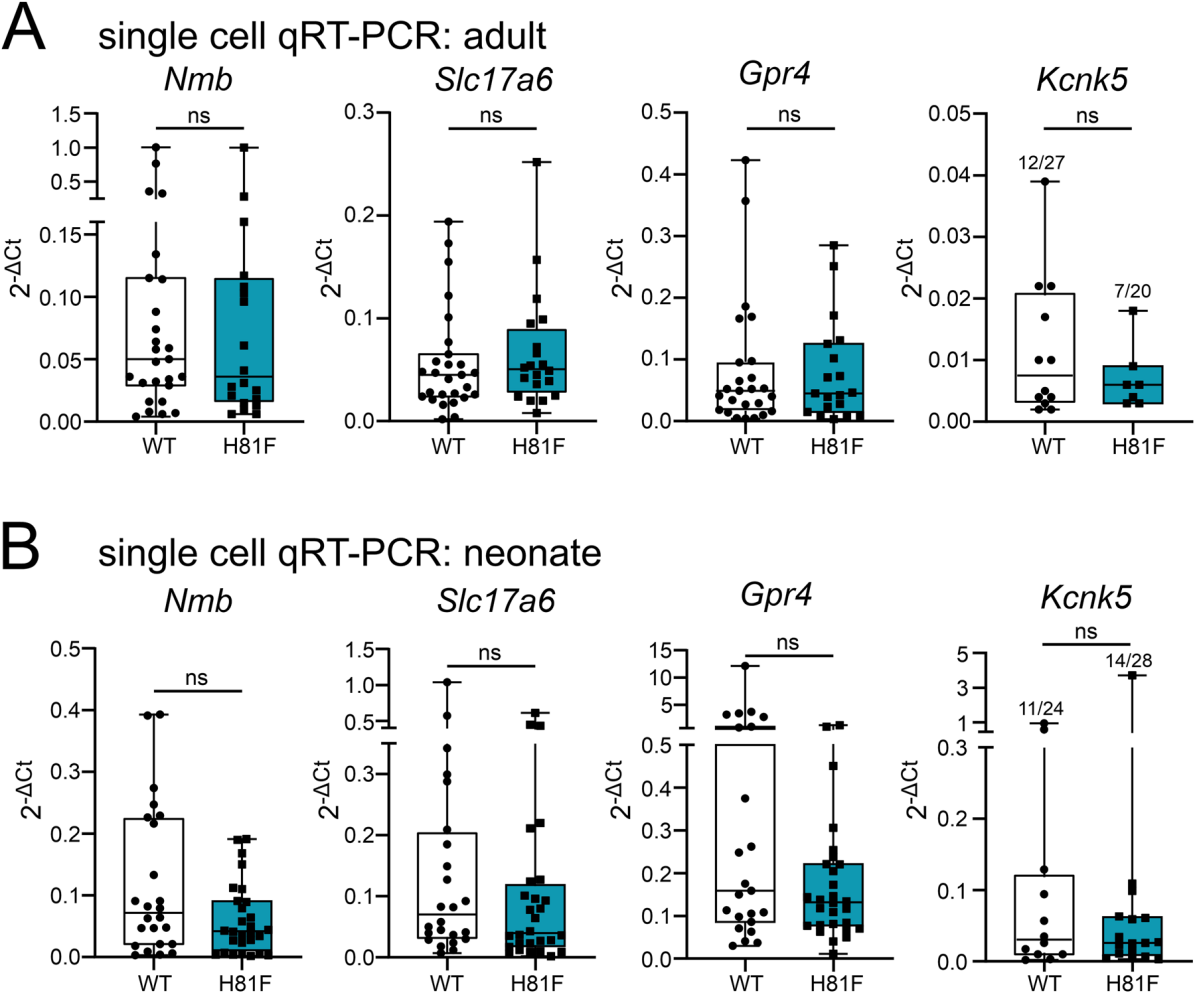
Expression levels of key molecular markers are not different in RTN neurons from WT and GPR4(H81F) mice. ***A***, ***B***, Single-cell qRT-PCR results for RTN markers *Nmb* and *vGlut2* as well as for proton sensors *Gpr4* and *Kcnk5* from adult (***A***, P49–P109) and neonatal (***B***, P7–P11) RTN neurons (adult: WT Jx99, *N* = 4; *n* = 27; H81F-Jx99, *N* = 3; *n* = 20; neonate: WT Jx99, *N* = 3; *n* = 24; H81F-Jx99, *N* = 3; *n* = 28; unpaired *t* test).

We also harvested individual GFP-labeled RTN neurons from acute brainstem slices obtained from WT Jx99 and H81F-Jx99 mice for sc-qPCR. We used expression of *Nmb* and *VGlut2* (i.e., *Slc17a6*) to verify that the cells were indeed RTN neurons and found that there was no difference in transcript levels for either *Gpr4* or TASK-2 (*Kcnk5*) in either neonatal or adult RTN neurons obtained from Jx99 or H81F-Jx99 mice (Fig. 6). Note that *Kcnk5* was detected in only approximately half of the RTN neurons, less than the expected based on previous scRNA-Seq or RNAscope analyses (e.g., >80%; Shi et al., 2017); nevertheless, this was the case for both Jx99 and H81F-Jx99 mice, at both developmental stages, suggesting a higher detection threshold for *Kcnk5* with this multiplexed sc-qPCR assay than with scRNA-Seq or RNAscope (Shi et al., 2017). Collectively, these data indicate that introducing a GPR4(H81F) substitution in mice reduces CO_2_-stimulated breathing along with the sensitivity of RTN neurons to changes in CO_2_/H^+^, without affecting the number or distribution of RTN neurons, expression of GPR4 or TASK-2, or basic neuronal excitability.

### CO_2_-stimulated breathing and CO_2_ sensitivity of RTN neurons is reduced in GPR4(H167F) mice

**Figure 7.**
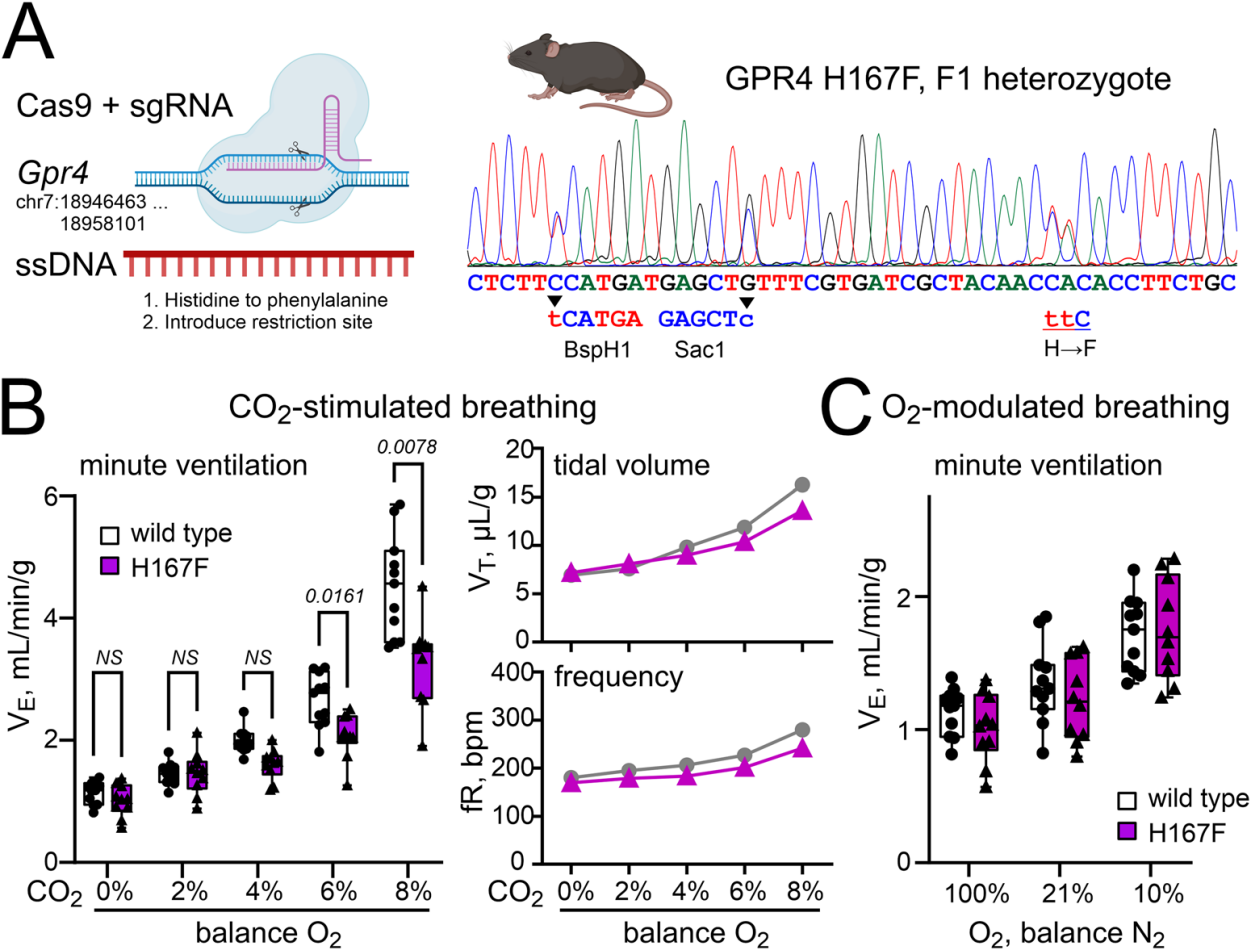
Mutation of alternative pH-sensing residue (His167) in GPR4 blunts CO_2_-stimulated breathing. ***A***, Sanger sequencing trace from an F1 heterozygote showing targeted alterations in *Gpr4* genomic sequence after CRISPR/Cas9 editing, with substitution of Phe for His167 and introduction of silent BspHI and SacI restriction sites. ***B***, CO_2_-stimulated V_E_ (left), V_T_ (upper right) and fR (lower right) of GPR4(H167F) and WT control (*N* = 10 and 11, P55–P136) mice in response to increasing levels of inspired CO_2_ (balance O_2_). *P* displayed are from two-way RM ANOVA with Šidák's multiple-comparison test: *F*_(1,19) _= 13.03; *p* = 0.0019 for genotype and *F*_(4,76) _= 10.06; *p* < 0.0001 for CO_2_ exposure × genotype. ***C***, Minute ventilation of H167F and WT control mice during exposure to the indicated levels of inspired O_2_. Two-way RM ANOVA analysis with Šidák's multiple-comparison test: *F*_(1,19) _= 0.4037; *p* = 0.5328 for genotype and *F*_(2,38) _= 1.823; *p* = 0.1754 for O_2_ exposure × genotype.

**Figure 8.**
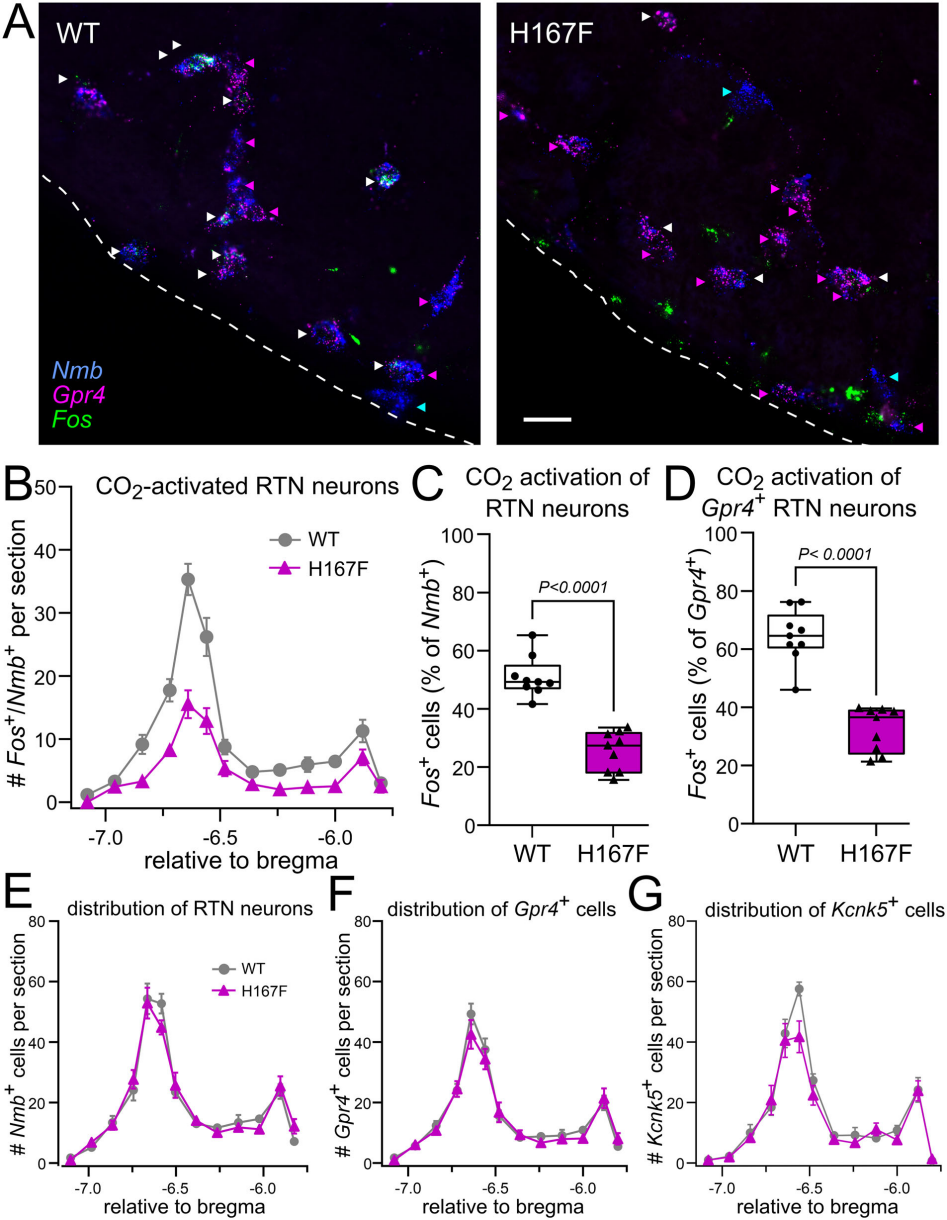
CO_2_-stimulated RTN neuron activation in vivo is blunted in GPR4(H167F) mice. ***A***, Representative RNAscope images for *Nmb*, *Gpr4*, and *Fos*. CO_2_-activated RTN neurons (*Nmb*^+^/*Gpr4*^+^/*Fos*^+^) are denoted with white arrowheads; *Gpr4*-expressing RTN neurons (*Nmb*^+^/*Gpr4*^+^) not activated by CO_2_ (i.e., *Fos*-negative) are denoted with magenta arrowheads; blue arrowheads denote *Nmb*^+^ cells expressing neither *Gpr4* nor *Fos*. ***B***, Distribution of CO_2_-activated RTN neurons (*Nmb*^+^/*Fos*^+^) throughout the rostrocaudal extent of the RTN in WT and H167F mice (mean ± SEM). ***C***, The total percentage of RTN (*Nmb*+) neurons expressing *Fos* (WT, *N* = 9; H167F, *N* = 9; P63–P190; unpaired *t* test). ***D***, The total percentage of GPR4-expressing RTN (*Gpr4*^+^ and *Nmb*^+^) neurons expressing *Fos*. ***E–G***, Rostrocaudal distribution of RTN neurons (*Nmb*^+^, ***E***), and of *Gpr4*^+^ (***F***) and *Kcnk5*^+^ (***G***) RTN neurons in WT and H167F mice [mean ± SEM, (***E***, ***F***) *N* = 9 and 10; (***G***) *N* = 6 and 7, unpaired *t* test].

We next considered the possibility that the observed CO_2_ sensing deficits might be specific to the H81F mutation, perhaps interfering with GPR4 function in a manner independent of effects on pH sensitivity. In order to address this possibility, we introduced another pH-desensitizing mutation, His167Phe, using a CRISPR/Cas9 strategy similar to that utilized for the H81F knock-in animals (Fig. 7*A*); for these GPR4(H167F) mice, the genetic substitution was made on a C57BL/6 background. As observed in the H81F animals, H167F mice also displayed a blunted HCVR relative to their WT control littermates (Fig. 7*B*), although the effect was not as pronounced (Δ*V_E_* in 8% CO_2_ reduced by ∼33%, from 3.4 ± 0.2 ml/min/g to 2.3 ± 0.2 ml/min/g; *N* = 11 and 10; *p* = 0.0025); again, there was no difference in the hypoxic ventilatory response between WT and H167F mice (Fig. 7*C*). In addition, in comparison with control littermates, we observed a reduced proportion of CO_2_-activated *Fos*^+^ neurons in H167F mice for either the *Nmb*^+^ or the *Nmb*^+^/*Gpr4^+^* populations of RTN neurons (Fig. 8*A*–*D*) without any differences in the overall number or distribution of *Nmb*^+^-, *Gpr4*^+^-, or *Kcnk5*^+^-expressing cells throughout the rostrocaudal extent of the RTN (Fig. 8*E*–*G*). Thus, these data from H167F mice largely phenocopy results from the H81F mice and support the contention that pH sensing by GPR4 contributes to CO_2_-stimulated breathing and CO_2_ sensitivity of RTN neurons in mice.

### Concurrent knock-out of TASK-2 in addition to H81F mutation of GPR4 has no additive effect to blunt CO_2_ sensitivity or activation of RTN neurons

**Figure 9.**
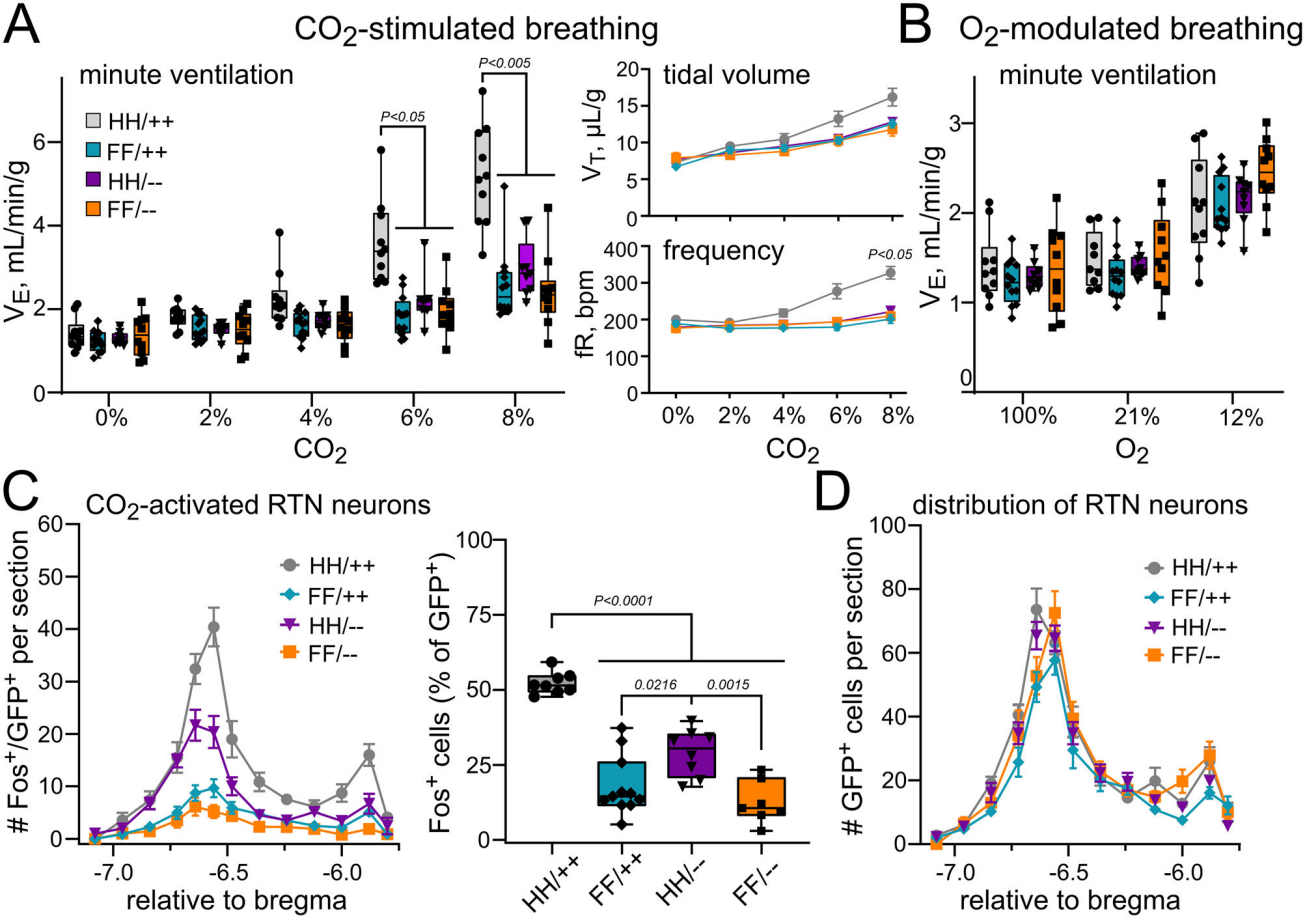
Concurrent deletion of TASK-2 in H81F-Jx99 mice does not further decrease the HCVR or RTN neuron activation. ***A***, Minute ventilation (V_E_), tidal volume (V_T_), and respiratory frequency (fR) in WT control (HH/++, *N* = 10, P68–P144), H81F (FF/++, *N* = 12, P61–P139), TASK-2 deleted (HH/−, *N* = 9, P61–P214), and double mutant H81F and TASK-2 deleted littermates (FF/−, *N* = 10, P61–P193) in response to increasing levels of inspired CO_2_ (balance O_2_). Two-way RM ANOVA analysis: V_E_, *F*_(3,35) _= 15.78; *p* < 0.0001 for genotype, and *F*_(12,140) _= 13.78; *p* < 0.0001 for CO_2 _× genotype; V_T_, *F*_(3,35) _= 2.802; *p* = 0.0541 for genotype, and *F*_(12,140) _= 4.337; *p* < 0.0001 for CO_2 _× genotype; fR, *F*_(3,35) _= 10.74; *p* < 0.0001 for genotype, and *F*_(12,140) _= 9.016; *p* < 0.0001 for CO_2 _× genotype. *P* values displayed are from Šídák's multiple-comparison test, for WT (HH/++) vs all other groups. ***B***, Minute ventilation of WT control (HH/++, *N* = 10), H81F (FF/++, *N* = 12), TASK-2 deleted (HH/−, *N* = 9), and double mutant H81F and TASK-2 deleted littermates (FF/−, *N* = 10) during exposure to the indicated levels of inspired O_2_. Two-way RM ANOVA analysis: *F*_(3,36) _= 1.271; *p* = 0.2988 for genotype, and *F*_(6,72) _= 3.259, *p* = 0.0068 for genotype × O_2_. (***C***, ***D***) Distribution (left) and total percentage (right) of Fos-immunoreactive GFP–labeled neurons and total number of GFP-expressing (***D***) neurons throughout the rostrocaudal extent of the RTN in WT control (HH/++, *N* = 7), H81F (FF/++, *N* = 11), TASK-2 deleted (HH/−, *N* = 6), and double mutant H81F and TASK-2 deleted littermates (FF/−, *N* = 7) after exposure in vivo to 12% CO_2_. The total percentage of RTN (GFP^+^) neurons expressing Fos for each group, analyzed by one-way ANOVA, *F* = 41.79; *p* < 0.0001; *p* values displayed are from Tukey's multiple-comparison test.

In previous work, we and others have found that the HCVR depends on both GPR4 and TASK-2—i.e., global deletion of either GPR4 or TASK-2 alone partially blunted the HCVR, whereas loss of both genes nearly eliminated CO_2_-stimulated breathing (Gestreau et al., 2010; Kumar et al., 2015; Guyenet et al., 2016). Here, to determine if loss of TASK-2 can eliminate the residual CO_2_ sensitivity observed in GPR4(H81F) animals, we generated mice homozygous for WT or H81F variants of GPR4 in the context of either intact or deleted TASK-2 genes (all on the Jx99 background). In comparison with control littermates (GPR4^+/+^;TASK-2^+/+^; HH/++), we found that CO_2_-stimulated breathing was reduced both in mice with GPR4(H81F) mutation alone (FF/++; by ∼65%, ΔV_E_ at 8% CO_2_: 3.8 ± 0.4 ml/min/g vs 1.3 ± 0.2 ml/min/g; *N* = 10 and 12) and in mice with TASK-2 deletion alone (HH/−; by ∼55%, 1.7 ± 0.3 ml/min/g; *N* = 9; Fig. 9*A*); this is consistent with the partial reduction in the HCVR noted in previous work from GPR4 knock-out and TASK-2 knock-out mice (Gestreau et al., 2010; Kumar et al., 2015). However, although CO_2_-stimulated breathing was reduced relative to controls in doubly mutated GPR4(H81F);TASK-2^−/−^ mice (FF/−; by ∼72%, 1.1 ± 0.3 ml/min/g; *N* = 9), there was no significant difference in the magnitude of the HCVR among any of the mutated mice. These data differ from those obtained previously with global knock-out mice, where combined deletion of GPR4 and TASK-2 further decreased CO­_2_ sensitivity compared with the loss of either gene alone (Kumar et al., 2015; Guyenet et al., 2016). As expected, there was no effect of any of these gene mutations on the hypoxic ventilatory response (Fig. 9*B*).

We also examined activation of RTN neurons by an acute CO_2_ challenge in vivo, again using Fos immunostaining as a proxy for neuronal activation, and with GFP immunoreactivity to label Phox2b-expressing RTN neurons in these singly and doubly mutated Jx99 mice. The number of Fos^+^ cells was lower across the rostrocaudal extent of the RTN in all mutant mice, compared with the WT control littermates (Fig. 9*C*). A slightly higher total number of Fos^+^ cells were obtained in RTN neurons from mice deleted only for TASK-2 on a WT GPR4 background (HH/−), as compared with the GPR4 single mutant (FF/++) or the GPR4 and TASK-2 double mutant mice (FF/−), but there was no difference between the latter two groups (Fig. 9*C*). We observed no difference in the number of GFP + RTN neurons among any of the groups (Fig. 9*D*). Thus, RTN neuronal activation by CO_2_ is disrupted by loss of function in both TASK-2 and GPR4, via the H81F mutation, with perhaps a smaller effect of TASK-2 deletion than GPR4 mutation on CO_2_-induced Fos expression.

### Blood chemistry is unaffected in GPR4(H81F) and GPR4(H167F) mice

GPR4 is expressed in relatively few neuronal populations outside the RTN (Kumar et al., 2015; Hosford et al., 2018; Gonye et al., 2024), but it is found in various peripheral tissues where it has been associated with several (patho)physiological processes, including acid–base regulation by the kidney (Yang et al., 2007; Sun et al., 2010, 2015; Cheval et al., 2021). We therefore performed arterial blood gas analysis on the different lines of mice to test for any chronic changes in blood gas concentrations or acid–base status (Tables 2, 3). In the GPR4(H81F) mice, relative to their WT littermates, we found a slight metabolic alkalosis with elevated HCO_3_^−^ levels. However, those values were not different from those of the WT or His-substituted littermates in either the GPR4(H81F)-Jx or the GPR4(H167F) mouse lines, and there were no differences in arterial HCO_3_^−^ PO_2_, PCO_2_, or lactate across any of these lines (Table 2). Animals with TASK-2 knock-out alone and in conjunction with GPR4(H81F) mutation tended to show a reduction in arterial HCO_3_^−^ and PCO_2_ with no change in arterial pH, but this effect seems to be driven by TASK-2 deletion as no deficits were noted in the GPR4(H81F) littermate controls (Table 3). Thus, we found no systematic differences in arterial blood gases or pH that can account for the effects of the GPR4 histidine substitutions on CO_2_-stimulated breathing or RTN neuronal CO_2_/H^+^ sensitivity.

**Table 3.** Arterial blood gas analysis of GPR4(H81F) mice on TASK-2^+/+^ and TASK-2^−/−^ background

Genotype	GPR4^81H/H^;TASK2^+/+^ (10)	GPR4^81F/F^;TASK2^+/+^ (9)	GPR4^81H/H^;TASK2^−/−^ (8)	GPR4^81F/F^;TASK2^−/−^ (9)
pH	7.40 ± 0.01	7.41 ± 0.01	7.35 ± 0.03	7.37 ± 0.02
PCO_2_ (mmHg)	27.1 ± 1.2	29.0 ± 1.2	23.5 ± 1.3	20.8 ± 1.2**
$\mathrm{HC}O_{3}^{-}$ (mM)	16.5 ± 1.0	18.2 ± 0.8	12.8 ± 0.6^†^	12.3 ± 1.0*
PO_2_ (mmHg)	94.5 ± 2.2	93.9 ± 2.7	101.0 ± 1.2	100.4 ± 2.2
lactate (mM)	5.2 ± 0.9	5.0 ± 0.8	6.0 ± 0.7	5.5 ± 0.7

Statistical analysis by one-way ANOVA with Dunnett’s multiple-comparison test comparing mutant/knock-out line to WT controls.

** *p* = 0.0018.

* *p* = 0.0141.

† *p* = 0.0035.

## Discussion

To delineate the sensory component of a homeostatic neural reflex pathway, it is necessary to identify both the constituent cells and the relevant molecular transducers. Among the different cell types implicated in mediating the HCVR (Gonye and Bayliss, 2023), it is now clear that RTN neurons play a prominent role (Souza et al., 2023), with important contributions from GPR4, a pH-sensitive receptor expressed in RTN neurons (Kumar et al., 2015). In this work, we used multiple lines of mice carrying different pH-insensitive GPR4 mutations—GPR4(H81F) or GPR4(H167F)—to provide evidence that it is the intrinsic pH sensitivity of GPR4, per se, that accounts for its effects on CO_2_/H^+^ sensitivity of RTN neurons and CO_2_-stimulated breathing. Thus, the HCVR was blunted and CO_2_/H^+^-mediated activation of RTN neurons was reduced in GPR4(H81F) or GPR4(H167F) mice. These effects could not be attributed to differences in the numbers or distribution of RTN neurons, to altered RTN expression of GPR4 (or the alternative proton detector, TASK-2), or to changes in basal excitability of RTN neurons in brainstem slices. Collectively, these data indicate that GPR4 is indeed a relevant and direct molecular CO_2_/H^+^ detector for RTN neurons, the HCVR and homeostatic regulation of CO_2_.

In agreement with previous work based on GPR4 knock-out mice (Kumar et al., 2015), we found that CO_2_/H^+^-stimulated RTN activation was diminished in vivo (decreased Fos expression) and in brain slices (decreased pH-dependent firing) in pH-insensitive GPR4 mutant mice. Moreover, we previously showed that viral-mediated reexpression of WT GPR4 in the RTN in the context of a global knock-out is sufficient to rescue deficits in CO_2_-stimulated breathing (Kumar et al., 2015). These observations implicate the RTN as a primary locus of action for GPR4 in the HCVR. Nevertheless, expression of the His-substituted GPR4 variants was not restricted to RTN in these mice, and it is therefore possible that some component of the blunted HCVR is due to disrupted GPR4-mediated CO_2_/H^+^-sensitivity in other GPR4-expressing cell groups. Of note in this regard, GPR4 expression is evident in both peripheral respiratory chemoreceptors (i.e., carotid body; Kumar et al., 2015) and other central chemoreceptors (i.e., serotonergic caudal raphe nuclei; Kumar et al., 2015; Hosford et al., 2018; Gonye et al., 2024). A contribution from carotid bodies seems unlikely since our plethysmography experiments are performed under hyperoxic conditions intended to silence peripheral chemoreceptors (Pepper et al., 1995) and because earlier work showed that inhibition of the HCVR in global GPR4 knockouts was retained after carotid body denervation (Kumar et al., 2015). For serotonergic raphe neurons, recent intersectional genetic approaches revealed that respiratory chemosensitivity is restricted to an Egr1-Pet1-expressing subset of medullary raphe cells (Brust et al., 2014; Hennessy et al., 2017; Okaty et al., 2019) even as GPR4 is expressed at moderate-to-high levels in serotonergic neurons of all brainstem raphe cell groups (Hosford et al., 2018; Gonye et al., 2024). In addition, we found here that the modest CO_2_-stimulated Fos expression in raphe neurons is unaffected in GPR4(H81F) mice, as also observed earlier in global GPR4 knock-out mice (Kumar et al., 2015). These observations do not support a GPR4 contribution to raphe neuron-mediated respiratory chemosensitivity, but a more direct test could be obtained from electrophysiological analysis of raphe neurons from GPR4(HF) mice, specifically from the Egr1-Pet1-expressing subset of serotonergic cells (Brust et al., 2014).

We found that mice with GPR4(H81F) or GPR4(H167F) substitutions, expressed in the context of multiple genetic backgrounds, displayed reduced CO_2_-evoked (Fos) activation of RTN neurons and CO_2_-stimulated breathing in vivo. The effects were somewhat less pronounced in the H167F line. However, it does not appear that differences in effect sizes between H81F and H167F mouse lines can be explained by the properties of the mutated receptors themselves, at least as measured by cAMP assays in transfected HEK293T cells. In that system, the H167F variant showed somewhat lower overall expression with generally equal surface expression, while both receptor mutants showed a similar reduction in cAMP production albeit with the H167F variant slightly more affected at the more acidic end of the relevant pH range, i.e., pH 7.6 to pH 7.2 (Guyenet et al., 2005). There are multiple potential reasons for imperfect transformation of mutational effects on GPR4-cAMP signaling characteristics in the HEK293T cell reconstitution system to a neuronal and behavioral phenotype. For example, there are well known and complex interactions between agonist affinity and agonist efficacy, with both strongly influenced by receptor expression levels, G-protein binding and the assay system employed (Kenakin, 2005; Strange, 2008). In this case, GPR4 expression may be different in neurons vs HEK293T cells (Hosford et al., 2018 for effects of GPR4 levels on pH sensitivity); receptor coupling mechanisms that modulate RTN activity may involve alternative G-protein–second messenger systems (e.g., Gαq-PLC rather than cAMP; Tobo et al., 2007; Liu et al., 2010); or small variations in constitutive activity of the mutated receptors may provoke different offsetting compensatory changes in the network. Alternatively, the modest differences in whole animal CO_2_ sensitivity observed between H81F and H167F mouse lines may reflect the different genetic background strains of the lines. That is, the H81F line was generated on a mixed B6SJLF1/J background that is at the low end of CO_2_ sensitivity, whereas the H167F line was created on a C57BL/6J background that has a particularly robust HCVR. Thus, it may be the case that the “hypercapnic high responsive” C57BL/6J strain is less susceptible to inhibition by GPR4 mutations than the “hypercapnic low responsive” SJL/J strains (Tankersley et al., 1994). If this is the case, there may be subtle differences between effects of the GPR4(H167F) mutation and GPR4 deletion since previous results from global GPR4 knock-out mice were obtained with C57BL/6J and Balb/c mice (Kumar et al., 2015), both of which are “hypercapnic high responsive” (Tankersley et al., 1994).

Despite the modest differences discussed above, we obtained substantively similar results in H81F and H167F knock-in animals, consistent with the interpretation that this reflects the common effect of those individual point mutations to disrupt proton-dependent activation of GPR4 (Liu et al., 2010). However, it remains formally possible that these two mutations instead disrupt receptor activation by some native agonist, perhaps in a pH-dependent manner. In this respect, miraculin is a fruit protein that converts from an antagonist at the hT1R2–hT1R3 sweet taste receptor to an agonist when exposed to acidic pH (Kurihara and Beidler, 1968; Koizumi et al., 2011; Misaka, 2013). Nonetheless, it is unlikely that such a pH-dependent agonist is required for proton-mediated GPR4 activation, which can be observed in cells after heterologous expression or inferred from GPR4-dependent effects in ex vivo preparations bathed in simple buffers (e.g., pH- and GPR4-dependent cAMP production or RTN neuronal firing in acidic HEPES-based buffers). Conversely, an alternative synthetic agonist that acts independently of pH would be a useful experimental tool to verify that these mutations do not have other unexpected inhibitory effects on receptor activation or downstream signaling.

In previous work, we showed that double knock-out of GPR4 and TASK-2 was similar to selective RTN ablation in essentially eliminating the HCVR (Kumar et al., 2015; Guyenet et al., 2016; Souza et al., 2023), whereas here we find that concurrent deletion of TASK-2 on the GPR4(H81F) mutant background did not further diminish the HCVR. It is possible that the residual pH sensitivity by GPR4(H81F) receptors, particularly at the highest CO_2_ levels, may contribute to the retained HCVR in the absence of TASK-2; even a diminished GPR4-mediated stimulation would not be available in the GPR4 knock-out. Likewise, CO_2_-induced activation of RTN neurons (Fos expression) was also equally and strongly reduced in H81F mice, irrespective of TASK-2 genotype, possibly because Fos activation is more easily disrupted than the HCVR, as also observed with GPR4 knock-out mice (Kumar et al., 2015). Finally, it is important to recognize that multiple alternative cellular mechanisms have been proposed to mediate respiratory chemosensitivity. These include effects of CO_2_ on astrocytes (e.g., via membrane depolarization or Cx26 carbamylation) or on other putative chemosensory cell groups, such as raphe, locus ceruleus, or orexin neurons (Gourine et al., 2010; Wenker et al., 2010; Wenker et al., 2012; Guyenet et al., 2016; van de Wiel et al., 2020; Dale, 2021; Guyenet and Bayliss, 2022; Gonye et al., 2024). It is possible that one or more of these may provide a greater contribution to the residual HCVR in these knock-in mice than was observed previously in the GPR4-TASK-2 double knock-out animals (Kumar et al., 2015).

Collectively, these data demonstrate that introducing either of the two single histidine mutations that disrupt the intrinsic pH sensitivity of GPR4 is sufficient to blunt CO_2_/H^+^ activation of RTN neurons and whole animal respiratory CO_2_ sensitivity, supporting the conclusion that the GPR4 is indeed a molecular proton sensor for the HCVR. GPR4 is expressed in additional anatomically and chemically distinct brain–body regions (Mahadevan et al., 1995; Hosford et al., 2018; Gonye et al., 2024), and these mice may also provide a useful tool to discover if direct proton sensing by this receptor contributes to additional behavioral (e.g., arousal, anxiety) or physiological (e.g., renal acid excretion) responses to CO_2_/H^+^.
